# Determining the Rate of Carbonic Anhydrase Reaction in the Human Brain

**DOI:** 10.1038/s41598-018-20746-x

**Published:** 2018-02-02

**Authors:** Shizhe Li, Li An, Qi Duan, Maria Ferraris Araneta, Christopher S. Johnson, Jun Shen

**Affiliations:** 1 National Institute of Mental Health, National Institutes of Health, Bethesda, MD USA; 2 National Institute of Neurological Disorders and Stroke, National Institutes of Health, Bethesda, MD USA

## Abstract

Carbonic anhydrase plays important role in life. This study sought to demonstrate the feasibility of detecting carbonic anhydrase activity in the human brain *in vivo*. After oral administration of [U-^13^C_6_]glucose, ^13^C saturation transfer experiments were performed with interleaved control spectra and carbon dioxide saturation spectra. Proton nuclear Overhauser effect pulses were used to increase signal to noise ratio; no proton decoupling was applied. Results showed that the ^13^C signal of bicarbonate was reduced by 72% ± 0.03 upon saturating carbon dioxide. The unidirectional dehydration rate constant of the carbonic anhydrase reaction was found to be 0.28 ± 0.02 sec^−1^ in the human brain. These findings demonstrate the feasibility of measuring carbonic anhydrase activity *in vivo* in the human brain, which makes it possible to characterize this important enzyme in patients with brain disorders.

## Introduction

Carbonic anhydrase (CA) is a ubiquitous, monomeric zinc metalloenzyme^1–4^ that catalyzes the reversible hydration of carbon dioxide and permits near equilibrium even at low substrate concentrations^1^. Because of carbon dioxide’s vital role, CA has many important functions, including gluconeogenesis, lipogenesis, tumorigenicity, and signal transduction^1,2,5–8^. In brain tissues, CA is primarily expressed in glial and choroid cells^9,10^. The lack of significant CA activity in neurons leads to the processing of carbon dioxide primarily in glial cells, which renders glial cells as sinks of carbon dioxide^11^. In addition, recent evidence suggests that under conditions of high neuronal activity, glial processing of carbon dioxide and energy transfer is coupled with high-affinity glutamate uptake by glia^11,12^.

CA inhibitors such as aromatic and heterocyclic sulfonamides have important clinical applications in the treatment of glaucoma^4^, epilepsy, and other neurological disorders^13,14^. CA activators, on the other hand, are used to manage conditions in which learning and memory are impaired, such as aging and Alzheimer’s disease^15^, as well as for the treatment of genetically inherited CA deficiencies^2,8,16^. Recent proteomic studies of brain disorders such as schizophrenia and major depression have revealed marked alterations in CA expression^17^. Interestingly, the well-known selective serotonin reuptake inhibitors fluoxetine, sertraline, and citalopram are strong CA activators^18^.

Considering the fundamental importance of CA, a noninvasive magnetic resonance spectroscopy (MRS) method capable of directly measuring the carbon dioxide–bicarbonate exchange rate catalyzed by CA in humans would clearly be valuable. Historically, *in vivo* enzyme-specific saturation transfer spectroscopy was limited to using ^31^P MRS for the study of creatine kinase and adenosine triphosphate (ATP) exchange reactions^19^. However, using rodent models, previous work from our laboratory demonstrated *in vivo* ^13^C saturation transfer effects catalyzed by aspartate aminotransferase, lactate dehydrogenase, malate dehydrogenase, and CA^20–23^. The present study used 7 Tesla MRS to examine the ^13^C saturation transfer effect of the carbon dioxide–bicarbonate exchange catalyzed by CA in human subjects. We also sought to quantify the pseudo first-order rate constant of this exchange in the dehydration direction (H^+^  + HCO_3_^−^ → CO_2_ + H_2_O) in the human brain.

## Results

**Figure 1:**
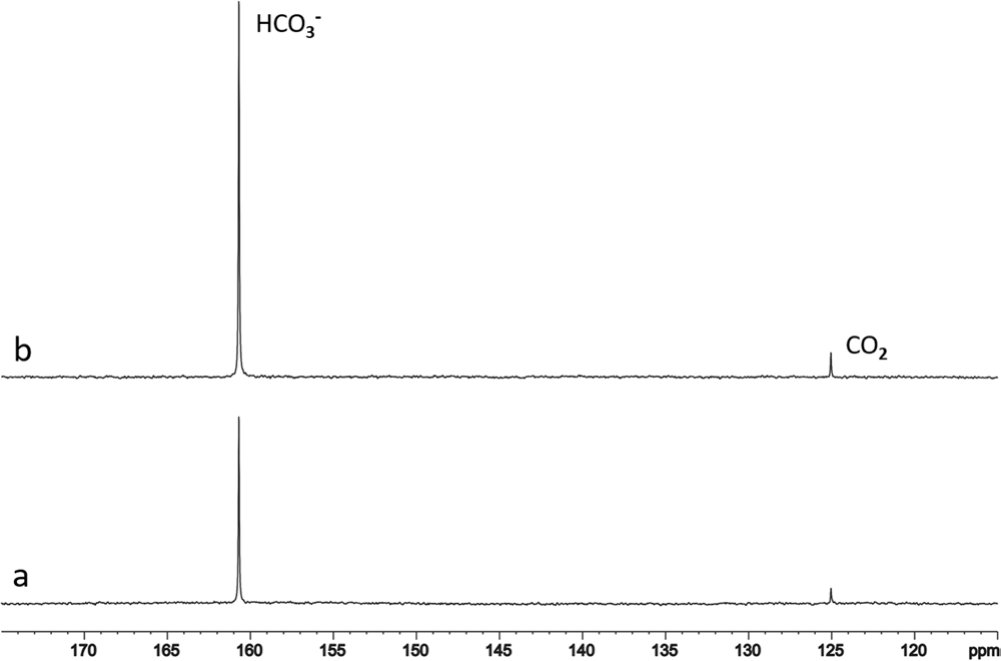
Nuclear Overhauser effect (NOE) demonstrated by the spectra acquired from phantom (20 mM NaH^13^CO_3_) (**a**) without proton NOE pulses, and (**b**) with NOE pulses. Signals of bicarbonate (160.7 ppm) and carbon dioxide (125.0 ppm) were increased by 101% and 60.5%, respectively. Repetition time (TR) = 35 s, number of average (NA) = 8, spectral width (SW) = 10 kHz, number of data points = 4096, line broadening (LB) = 4 Hz.

**Figure 2:**
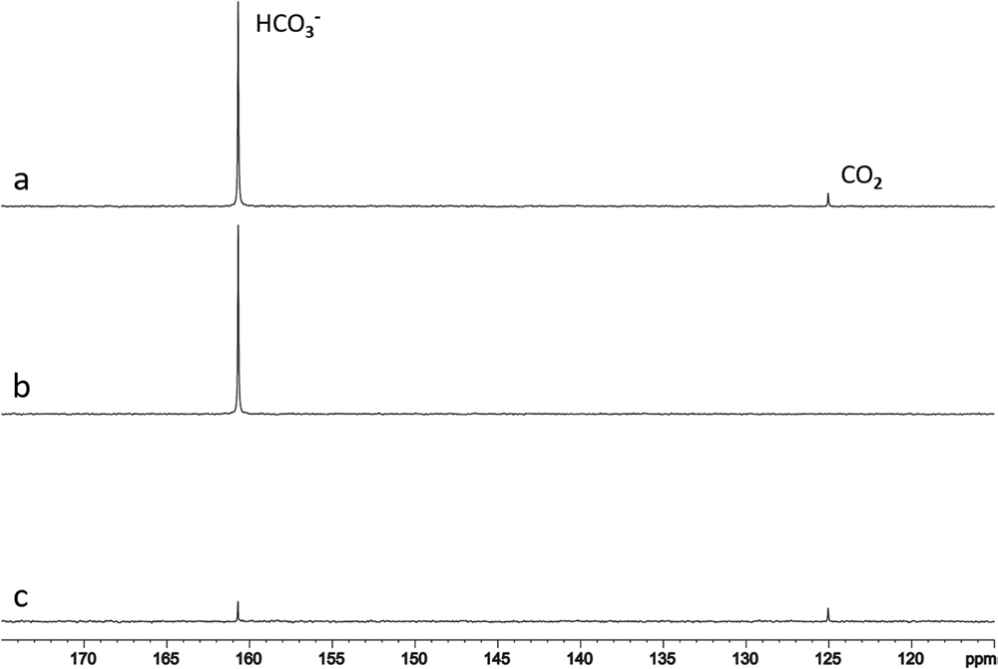
^13^C saturation transfer effect in the phantom due to exchange between carbon dioxide and bicarbonate in the absence of catalysis by carbonic anhydrase (CA). The control spectrum (a) was the same as the NOE-enhanced spectrum in Fig. 1. The bicarbonate signal was appreciably reduced upon radio frequency (RF) saturation of carbon dioxide (b). Note that the signal of carbon dioxide disappeared in (b). The difference spectrum (c) revealed an 8.2% decrease at ambient temperature and pH = 7.2 caused by the uncatalyzed exchange between carbon dioxide and bicarbonate in the phantom.

The results of phantom measurements are shown in Figs 1 and 2. Figure 1 compares nuclear Overhauser effect (NOE) enhancement of the bicarbonate and carbon dioxide signals while saturating protons at the resonance frequency of water using a phantom containing 20 mM NaH^13^CO_3_ (pH = 7.2). In Fig. 1, spectrum (a) was acquired without proton NOE pulses, and spectrum (b) was acquired with NOE pulses. Both bicarbonate (160.7 ppm) and carbon dioxide (125.0 ppm) signals were detected. At pH = 7.2, the spectrum was dominated by the bicarbonate signal, as expected from the Henderson–Hasselbalch equation[1]. When the two spectra were compared, signals of bicarbonate and carbon dioxide were increased by 101% and 60.5%, respectively.

The ^13^C saturation transfer effect of the uncatalyzed exchange between carbon dioxide and bicarbonate was investigated using the same phantom at ambient temperature. Figure 2 shows the observed ^13^C saturation transfer effect due to the exchange between carbon dioxide and bicarbonate in the absence of catalysis by CA. The control spectrum (Fig. 2a) was the same as the NOE-enhanced spectrum in Fig. 1. We found that the bicarbonate signal was appreciably reduced upon radio-frequency (RF) saturation of carbon dioxide (Fig. 2b), and that the signal of carbon dioxide disappeared (Fig. 2b). The difference spectrum revealed an 8.2% decrease at ambient temperature, and pH = 7.2 caused by the uncatalyzed exchange between carbon dioxide and bicarbonate in the phantom (Fig. 2c). The T_1_ of bicarbonate in the phantom was found to be 35 s, as measured using the equation for T_1_ calculation given by Xu and colleagues^22^. The same T_1_ was found with and without NOE enhancement. Using Eq. (1) (see Methods), the uncatalyzed unidirectional dehydration rate constant was 0.0058 sec^−1^.

**Figure 3:**
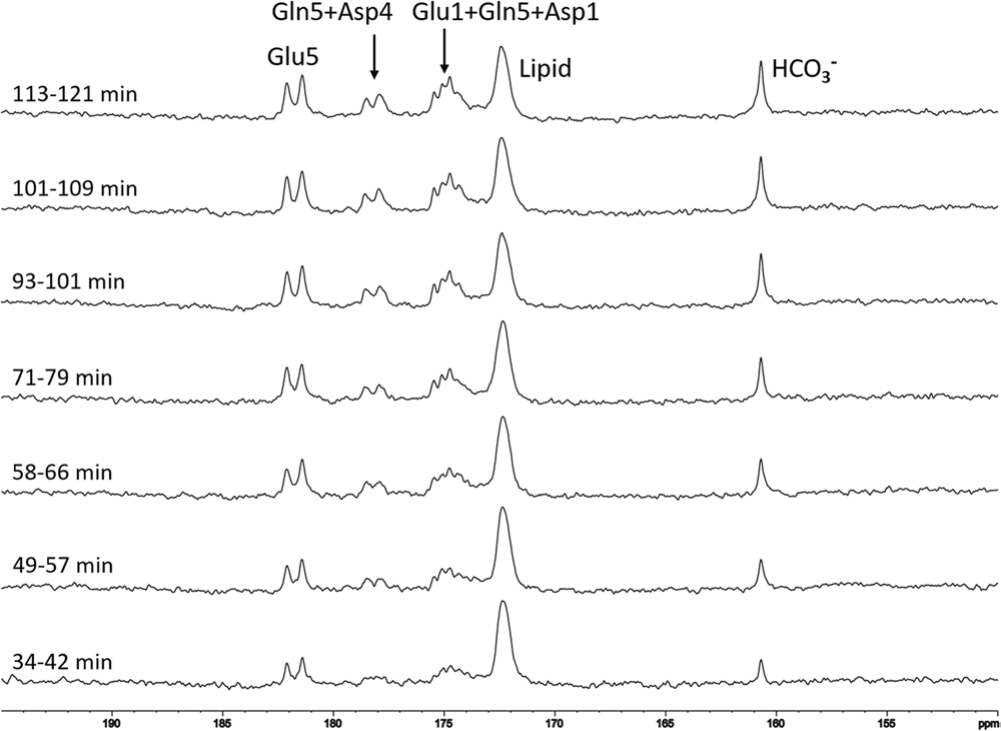
A typical time-course of control spectra from human brain after oral administration of [U-^13^C_6_]glucose without proton decoupling. Each spectrum was acquired with repetition time (TR) = 30 s, spectral width (SW) = 8 kHz, number of data points = 2048, number of average (NA) = 12, line broadening (LB) = 8 Hz. Natural abundance lipid carboxylic carbons (172.5 ppm), ^13^C-enriched glutamate C5 (Glu5, 182.0 ppm), glutamate C1 (Glu1, 175.4 ppm), glutamine C5 (Gln5, 178.5 ppm), glutamine C1 (Gln1, 174.8 ppm), aspartate C4 (Asp4, 178.3 ppm), and aspartate C1 (Asp1, 175.0 ppm) were observed.

**Figure 4:**
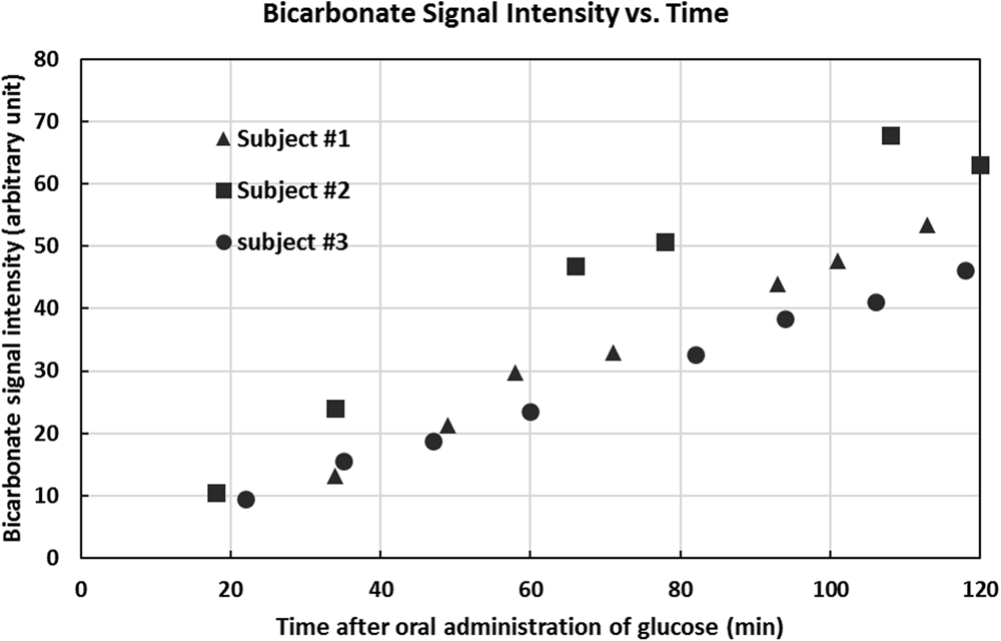
The signal intensities of bicarbonate from three subjects as a function of time. Although blood glucose levels from the subjects fluctuated during the scan time, the signals of bicarbonate from the three subjects increased consistently. The missing data points from the second subject was due to the patient’s requests to urinate.

During *in vivo* scans, blood samples were collected every 10 minutes to measure blood glucose concentrations. Although the total glucose reading fluctuated during the scan, signals of bicarbonate and other metabolites increased steadily and reached their approximate maxima at 100–120 minutes after oral administration (see Figs 3 and 4).

Saturation transfer effect was measured from three healthy volunteers. A typical time-course of control spectra from human brain after oral administration of [U-^13^C_6_]glucose without proton decoupling is shown in Fig. 3. The spectra of carboxylic/amide carbons with oral administration of [U-^13^C_6_]glucose were comparable to those with intravenous infusion of [2-^13^C]glucose^24^. A natural abundance of lipid carboxylic carbons (172.5 ppm), ^13^C-enriched glutamate C5 (Glu5, 182.0 ppm), glutamate C1 (Glu1, 175.4 ppm), glutamine C5 (Gln5, 178.5 ppm), glutamine C1 (Gln1, 174.8 ppm), aspartate C4 (Asp4, 178.3 ppm), and aspartate C1 (Asp1, 175.0 ppm) were observed (Fig. 3). Because of the large homonuclear ^13^C-^13^C coupling (51 Hz) due to the administration of uniformly ^13^C-labeled glucose, the signal from glutamate C5 appeared as a doublet. Signals from other carboxylic/amide carbons also experienced homonuclear splitting as well as line-broadening due to long-range heteronuclear couplings, and their resonances overlapped each other in the spectrum. No signal from carbon dioxide was detected *in vivo*^25^. Because all six ^13^C labels of [U-^13^C_6_]glucose contribute to the final bicarbonate ^13^C signal—due to the actions of pyruvate dehydrogenase, isocitrate dehydrogenase, and α-ketoglutarate dehydrogenase—the ^13^C signal of bicarbonate became much stronger than that acquired in the previous study with intravenous infusion of [2-^13^C]glucose^26^.

The signal intensities of bicarbonate from the three subjects as a function of time is given in Fig. 4 showing that the bicarbonate signals increased approximately monotonically. For the three subjects, the same time dependencies were also consistently observed for signals from the other metabolites (glutamate C1 and C5, glutamine C1 and C5, and aspartate C1 and C4).

**Figure 5:**
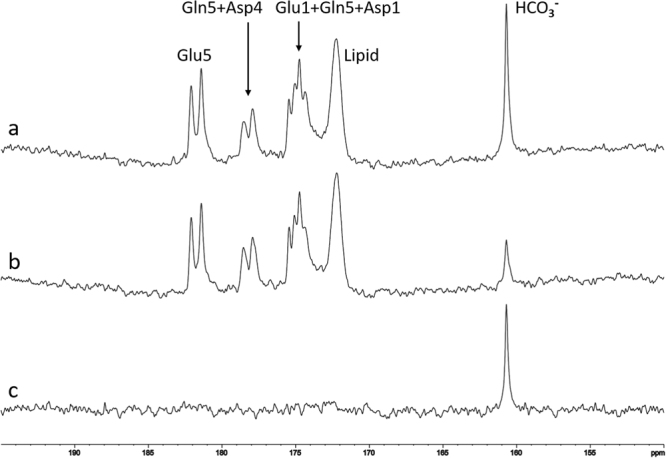
The saturation transfer effect catalyzed by carbonic anhydrase (CA) in the human brain. The spectra were obtained from a single subject, measured between 110 and 122 minutes after oral administration of [U-^13^C_6_]glucose. The top spectrum (a) is the control spectrum with ^13^C irradiation at 228 ppm; the middle spectrum (b) is the one obtained upon saturation of carbon dioxide at 125.0 ppm; the bottom spectrum (c) is the difference spectrum. Data acquisition parameters were the same as those in Fig. 4. The signal intensity of bicarbonate was reduced by 72% ± 0.03 (n = 3).

The spectra of ^13^C saturation transfer experiments obtained from a single subject, measured between 110 and 122 minutes after the oral administration of [U-^13^C_6_]glucose, is shown in Fig. 5. The top spectrum (Fig. 5a) is the control spectrum with the ^13^C irradiation at 228 ppm, the middle one (Fig. 5b) is the spectrum obtained upon the saturation of carbon dioxide at 125.0 ppm, the bottom one (Fig. 5c) is the difference spectrum. Based on the results of the three subjects, the signal intensity of bicarbonate was found to be reduced by 72% ± 0.03 (mean ± SD, n = 3) due to saturating carbon dioxide.

**Figure 6:**
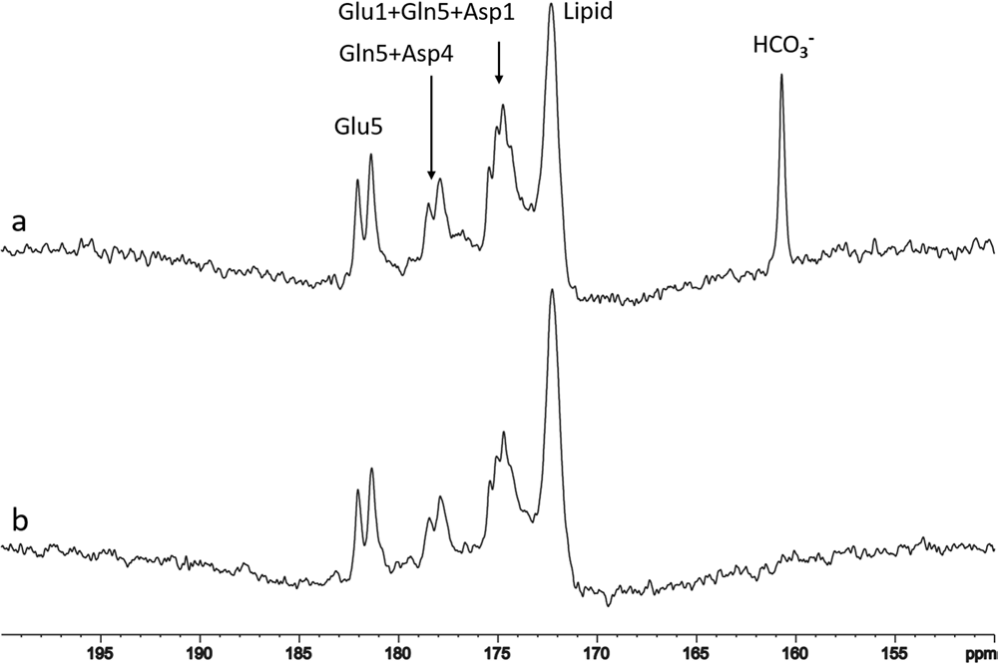
Results of *in vivo* T_1_ measurement of ^13^C bicarbonate using the ^13^C metabolite null method. Trace (a) was the non-inverted spectrum, and trace (b) was the inverted spectrum with null signal at recovery time of 7.0 s, repetition time (TR) = 55 s, number of average (NA) = 8, spectral width (SW) = 2 kHz, number of data points = 4096, line broadening (LB) = 4 Hz.

T_1_ of bicarbonate resonance was measured from three different subjects using the inversion recovery method. The results of *in vivo* T_1_ measurements of ^13^C bicarbonate using the ^13^C metabolite null method were plotted in Fig. 6. Trace (a) is the non-inverted spectrum and trace (b) is the inverted spectrum with null signal at a recovery time of 7.0 s. The measured T_1_ was 9.6 ± 0.3 s (mean ± SD, n = 3). Using Eq. 1 (see Methods), with the measured values of M^sat^/M^n^°^sat^ and the mean of T_1_ (9.6 s), the pseudo first-order rate constant for the unidirectional dehydration (k) in the human brain was determined to be 0.28 ± 0.02 sec^−1^ (n = 3).

## Discussion

This 7 Tesla MRS study is the first to demonstrate that CA activity can be detected *in vivo* in the human brain. Notably, although all of our experiments were performed using a ^13^C saturation transfer technique at 7 Tesla, the intense labeled bicarbonate ^13^C signal observed after administration of uniformly ^13^C-labeled glucose, and the dramatic change in the bicarbonate signal (72% reduction) observed in conjunction with carbon dioxide saturation, suggests that this technique could be readily extended to lower and more clinically relevant field strengths such as 3 Tesla.

The ^13^C signal of bicarbonate at 160.7 ppm has been observed in the human brain when detecting carboxylic/amide carbons with infusion of [1-^13^C]glucose^27^, [1-^13^C]acetate^28^, and [2-^13^C]glucose^26^ infusions. Although the concentration of bicarbonate in the brain under normal physiological conditions is relatively high (>20 mM)^29^,  previous reports noted that the observed ^13^C signal intensity of bicarbonate was relatively weak compared with the signal intensity of many other ^13^C-labeled metabolites. There may be several reasons for this. First, to acquire carboxylic/amide carbons from amino acids, the ^13^C excitation pulse may be optimally placed on the carboxylic/amide carbons near 180.0 ppm. If the bandwidth of the ^13^C excitation pulse is not wide enough, particularly for high field experiments, ^13^C-labeled bicarbonates may not be appropriately excited. Second, when a single ^13^C-labeled substrate is administered in ^13^C MRS studies, only one ^13^C label per substrate molecule can be incorporated into bicarbonate. Therefore, the fractional enrichment of ^13^C-labeled bicarbonate is relatively low. To increase the bicarbonate signal for the present saturation transfer experiments, we: (a) placed the center frequency of the ^13^C excitation pulse at the bicarbonate frequency of 160.7 ppm, (b) used a TR of 30 s for the saturation transfer measurements and 55 s for the T_1_ measurements, and (c) administered [U-^13^C_6_]glucose to the subjects so that all six ^13^C labels of the substrate would contribute to the final bicarbonate ^13^C signal via the actions of pyruvate dehydrogenase, isocitrate dehydrogenase, and α-ketoglutarate dehydrogenase. As a result of these approaches, the bicarbonate signal was increased significantly. Coupled with the very large saturation transfer effect catalyzed by CA, the overall sensitivity of this experiment was uniquely high for ^13^C MRS.

Due to the rapid, CA-catalyzed exchange between carbon dioxide and bicarbonate, as well as the rapid exchange between the proton of bicarbonate and water, the small two-bond ^1^H-^13^C scalar coupling in bicarbonate is not observable even in the absence of heteronuclear decoupling in both phantom and *in vivo* scans. Therefore, proton decoupling was not applied in this study, which also helped to largely reduce RF deposition and the associated specific absorption rate. Results from the ^13^C-labeled bicarbonate phantom experiment (Fig. 1) demonstrated that the bicarbonate signal was increased by a factor of 2.0 due to the NOE via dipole-dipole interactions between bicarbonate ^13^C labels and surrounding protons. Interestingly, although carbon dioxide was not protonated at all, a highly significant (~60%) NOE enhancement of the carbon dioxide signal was also observed. This is likely due to a carry-over of the NOE effect from bicarbonate via interconversion between the two molecules. The magnitude of the NOE on bicarbonate found in this study is very similar to that observed in our previous 7 Tesla phantom study, where signals from glutamine and aspartate were increased by a factor of 2.3^24^. In short, we have successfully measured a large and quantifiable carbon saturation transfer effect in the human brain by combining multiple mechanisms to enhance the bicarbonate ^13^C signal with the relatively long ^13^C T_1_ of bicarbonate and the fast exchange between carbon dioxide and bicarbonate catalyzed by CA.

The present study further demonstrated that the ^13^C signal of carbon dioxide was detected in the phantom of ^13^C enriched bicarbonate phantom (Fig. 2), but not in human brain. There are several possible reasons for this. First, the concentration of dissolved free carbon dioxide gas in brain tissue is ~1 mM at normal physiological condition^29^, which makes it difficult to detect *in vivo* in the brain. Second, the intrinsic T_1_ of the unprotonated carbon dioxide is expected to be longer than that of the protonated bicarbonate. Third, detection of the carbon dioxide resonance *in vivo* could also be hampered by the off-resonance effect of the excitation pulse placed at the resonance frequency of bicarbonate. Because the pseudo first-order dehydration rate constant depends on M^sat^/M^nosat^ of bicarbonate and its T_1_ only, the concentration of carbon dioxide has no effect on its quantification. It should be noted that the concentrations of carbonic acid and carbonate were negligibly low at physiological (neutral) pH. They are also in rapid exchange with bicarbonate. Consequently, their *in vivo* ^13^C MRS signals were not observed either.

Following traditional saturation transfer techniques, the frequency of the control irradiation in our scans was initially set at 196.4 ppm, the same frequency difference from bicarbonate (35.7 ppm) but on the downfield side of the bicarbonate resonance. However, at this frequency setting, a small amount of residual signal was observed in the difference spectra in the region from 174.0 ppm to 176.0 ppm, which is dominated by glutamate C1 resonance. The residual signal can be explained by partial saturation of the C1 resonance of α-ketoglutarate hydrate, possibly bound to larger molecules. However, the exact source of this residual signal is unknown. After the control pulse was slightly moved downfield to 228.0 ppm, the residual signal in the difference spectra disappeared, and this shifted frequency was used for all control scans. Nevertheless, this small frequency shift should not have affected the bicarbonate signal because: (1) previous *in vivo* experiments repeatedly showed the nonexistence of nonspecific background magnetization transfer among the very diluted ^13^C spins^22,23^ and (2) the large frequency separation between the control frequency (228.0 ppm) and the resonance frequency of bicarbonate (160.7 ppm) ruled out any RF spillover effects commonly seen in ^31^P saturation transfer experiments.

^13^C-labeled substrates are commonly infused into human subjects via two intravenous (IV) insertions on a subject’s forearms; one is used to monitor blood glucose levels and the other is used to infuse the ^13^C-labeled substrates. There are many advantages of using intravenous glucose infusion including increased delivery of glucose into the brain. However, placing two IV catheters may increase the chance of causing pain to the subject and other adverse effects, including possible clogging of the infusion line that may interrupt the infusion process and cause swelling. In this study, glucose solution was administered orally^30–32^ so that only one IV line was needed to monitor blood glucose levels. With the dosage of 0.75 grams 99% ^13^C enriched glucose per kg of body weight, the amount of the glucose drunk by the subjects was between 40–50 grams, depending on body weight. The amount of glucose orally administrated into the subjects is very similar to that administrated via IV infusion in our previous infusion protocol^24,26^. The metabolism of ^13^C-labeled metabolites is slower with oral administration due to delayed glucose uptake by brain, but the peak fractional enrichment of ^13^C-labeled metabolites is not smaller than that associated with the intravenous infusion method if sufficient time is allowed^32^. In our study, larger fluctuations of blood glucose levels were observed (data not shown). The use of a longer catheter line in the 7 T magnet and the increased amount of “dead” blood volume (therefore dilution of blood by saline) may have contributed to this observation. Nonetheless,^13^C enrichment of the brain metabolites increased steadily over the time course (Fig. 3). During the interleaved data acquisition (TR = 30 s), each pair of control spectrum and carbon dioxide-saturated spectrum was taken in one minute. Within one minute, the fractional enrichment of bicarbonate could be considered stable because of the large dampening effect of various decarboxylation reactions, including the tricarboxylic acid cycle with a time constant of approximately one hour. Therefore, the spectroscopically observed saturation transfer observed here is considered very accurate.

Previous studies reported that the T_1_ of bicarbonate was 11.8 s in the anesthetized rat brain at 11.7 Tesla^25^. To accurately measure T_1_ of bicarbonate in human brain at 7 Tesla, a TR of 55 s was applied to satisfy the condition of TR ≥ 5 T_1_. Our measurement (n = 3) showed that T_1_ of bicarbonate in human brain at 7 Tesla was 9.6 ± 0.3 (n = 3). The shorter T_1_ found in this study may be attributable to generally faster relaxation in the more iron-rich human brain.

Finally, although only the cerebral carbon dioxide-bicarbonate exchange catalyzed by CA was investigated in this study, it is expected that the *in vivo* ^13^C saturation transfer spectroscopy method can also be used to study the CA-catalyzed carbon dioxide-bicarbonate exchange in other organs/tissues because of the wide distribution of CA, carbon dioxide, and bicarbonate.

In summary, this study demonstrated that bicarbonate and carbon dioxide are in rapid exchange in the human brain under catalysis by CA. The exchange is sufficiently rapid *in vivo* to lead to a large and quantifiable ^13^C saturation transfer effect. The signal intensity of bicarbonate was significantly boosted by administering [U-^13^C_6_]glucose. Because the ^13^C bicarbonate signal was not overlapped by that of scalp lipids, spatial localization methods such as SPLASH^33^ may be used to extract CA activity from a predefined brain region with natural anatomical boundaries.

## Methods

*In vivo* ^13^C MRS experiments were performed on a Siemens Magnetom 7 Tesla scanner (Siemens Healthcare, Erlangen, Germany) with VB17 software. The in-house built RF coil assembly comprised a circular ^13^C coil (diameter = 7 cm) and a quadrature half-volume proton coil. They were mounted on three vertically stacked semi-cylindrical plastic tubes. Each proton loop had a single-tuned ^1^H cable trap constructed using the RG-316 cable. A ^13^C/^1^H dual-tuned cable trap, built inside a shielded box, was connected to the ^13^C coil. At 75 MHz, the isolation between the ^13^C coil and both proton loops was −38 dB. At 300 MHz, the isolation between the two proton loops and between the ^13^C coil and both proton loops was −20 and −40 dB, respectively. The coil assembly was connected to the 7 Tesla scanner via an interface box (Quality ElectroDynamics, Mayfield Village, Ohio, USA) that included transmit-receive switches, pre-amplifiers, and RF filters for both channels, as well as a quadrature combiner for the proton channel. Details of the coil design, fabrication, its RF safety evaluation and positioning of human subjects were described in a previous publication from our laboratory^24^.

Healthy human subjects (n = 6) were recruited and consented using the procedures approved by the Institutional Review Board (IRB) of the National Institute of Mental Health (NIMH). The administration of ^13^C enriched glucose solution has been approved by IRB-NIMH and the National Institutes of Health (NIH) Clinical Center Pharmacy Department. All experimental protocols and methods were performed in accordance with the guidelines and regulations of NIH MRI Research Facility. Subjects fasted at least 12 hours before MRS scans. A solution of 20% w/w 99% enriched [U-^13^C_6_]glucose was orally administrated to subjects at a dose of 0.75 g [U-^13^C_6_]glucose per kg of body weight^32^ before initiation of ^13^C MRS scans. One antecubital vein was cannulated for withdrawing blood every 10 minutes to monitor blood glucose levels during the entire study.

A gradient-echo based 3-plane localizer was first used to properly position each subject. Static magnetic (B_0_) field shimming was performed using Siemens 3D Shim tool that included full first- and second-order shims, and axial terms of third-order shims (Z^3^, Z^2^X, Z^2^Y, Z(X^2^ − Y^2^)). A voxel of 5 × 5 × 5 cm^3^ cube was selected to perform B_0_ shimming in the occipital lobe directly above the ^13^C surface coil. A point resolved spectroscopy (PRESS) sequence^34^ was used to acquire water spectrum from the shimmed voxel in order to evaluate the convergence of shim adjustments. Typical water linewidth (full width at half maximum) from the 125 cm^3^ cubic voxel was 14.8 ± 2.1 Hz. The procedures used to calibrate RF transmit power for the proton and ^13^C coils have been previously described^24^. Briefly, RF power in the proton channel was calibrated *in-situ* using a two-dimensional stimulated echo method that generates a one-dimensional profile along the y-column. The transmit voltage of a proton excitation pulse was adjusted to generate a 180^o^ null along the y-direction. This voltage was used as a reference for NOE pulses. The RF power of the ^13^C coil was calibrated using a phantom of a three-liter cylindrical bottle filled with distilled water and 6 grams NaCl. ^13^C transmit voltage for a 500 μs 90° hard pulse was determined using the maximum signal of [1-^13^C]glucose in a small sphere inside the cylinder.

The following parameters were used to acquire phantom ^13^C MRS: repetition time (TR) = 35 s, number of average (NA) = 8, spectral width (BW) = 10 kHz, number of data points = 4096, line broadening (LB) = 4 Hz.

*In vivo* ^13^C saturation transfer spectra were acquired from three healthy subjects using a modified Siemens free induction decay sequence. The sequence had an interleaved acquisition scheme: {control irradiation – bicarbonate excitation – acquisition} – {carbon dioxide saturation – bicarbonate excitation – acquisition}. The excitation hard pulse (0.25 ms) was placed at the resonance frequency of bicarbonate (160.7 ppm). Acquisition parameters were SW = 8 kHz, data points = 2048, acquisition time = 256 ms, and TR = 30 s. During the entire recycle delay of 30 s, a 50 ms pulse block was repeatedly executed. In each pulse block, a 1.0 ms proton hard pulse (120°) was applied first at water frequency to generate heteronuclear NOE, then a 48.0 ms continuous wave pulse (γB_1_ = 50 Hz) was used to saturate carbon dioxide at 125.0 ppm or for control irradiation at 228.0 ppm. No proton decoupling was applied. The average RF power was less than 2.8 W. Each interleaved dataset contained 24 spectra (NA = 24). Twelve odd-number spectra had control irradiation at 228.0 ppm, and 12 even-number spectra had a saturation pulse placed on carbon dioxide at 125.0 ppm.

Prior to performing *in vivo* studies, phantom experiments were conducted to evaluate NOE and the saturation transfer effects of the uncatalyzed exchange between carbon dioxide and bicarbonate. The phantom was a three-liter cylindrical bottle filled with distilled water and 6 g NaCl. The loading effect of this phantom is approximately the same as that of an adult human head based on the measurement of loaded Q-value of the coils. Inside the bottle, a 7-cm sphere containing 20 mM NaH^13^CO_3_ (pH = 7.2) was attached to the cylindrical surface of the bottle directly above the ^13^C coil. The interleaved ^13^C pulse sequence described above was used in the phantom experiments with the following changes: (a) the ^13^C excitation pulse was placed at the frequency of carbon dioxide (125.0 ppm), (b) TR = 35 s, NA = 16, and (c) control spectra were acquired without any ^13^C pre-irradiation.

The T_1_ of *in vivo* ^13^C bicarbonate signal is relatively long^25^. When bicarbonate is partially saturated due to insufficient TR, the pseudo first-order rate constant for unidirectional dehydration (H^+^  + HCO_3_^−^ → CO_2_ + H_2_O) can be calculated using the following equation [22]:

1 $$\frac{{M}^{sat}}{{M}^{nosat}}=\frac{1}{1+k{T}_{1}}\frac{1-{e}^{-TR(\frac{1}{{T}_{1}}+k)}}{1-{e}^{-TR/{T}_{1}}},$$

where M^sat^ is the bicarbonate ^13^C magnetization measured while saturating carbon dioxide, M^nosat^ is the bicarbonate ^13^C magnetization without saturation of carbon dioxide, TR is repetition time, T_1_ is the spin-lattice relaxation time of bicarbonate without saturation of carbon dioxide, and k is the pseudo first-order rate constant for the unidirectional dehydration reaction. *In vivo* T_1_ of bicarbonate was measured using the inversion-recovery null method without saturating carbon dioxide. A hyperbolic secant pulse (30 ms) was used to invert the bicarbonate ^13^C signal. For T_1_ measurements, TR was set to 55 s.

### Data availability statement

The data of this study are available from the corresponding author upon request.
